# Reviewed article: Limongi JE, Santos KAR, Perissato IL, Faria RM. Spatial analysis of granting of social security benefits to people with gastrointestinal Chagas disease in Brazil, 2004-2016: a time series study. Epidemiol Serv Saude. 2025:34;20240622.

**DOI:** 10.1590/S2237-9622025v34e20240622.a

**Published:** 2025-06-13

**Authors:** Valéria de Campos Orsi

**Affiliations:** Universidade da Sorocaba

**Table:** 

Rating	Excellent	Good	Average	Below average	Poor
Interest	✓				
Quality	✓				
Originality	✓				
Overall	✓				

**Table:** 

Questionnaire	Yes	No	Not applicable
Does the manuscript contain new and significant information to justify publication?	✓		
Does the Abstract (Summary) clearly and accurately describe the content of the article?	✓		
Is the problem significant and concisely stated?	✓		
Are the methods described comprehensively?	✓		
Are the interpretations and conclusions justified by the results?	✓		
Is adequate reference made to other work in the field?	✓		

## Manuscript Structure

Length of article is: Adequate

Number of tables is: Too few

Number of figures is: Adequate

Please state any conflict(s) of interest that you have in relation to the review of this paper (state “none” if this is not applicable): None

## Comments to the Author

1. Os objetivos da pesquisa estão bem definidos e a hipótese é clara e testável.

O estudo apresenta justificativa e objeto claro.

2. O delineamento escolhido é adequado para responder à questão de pesquisa.

Sim.

3. Os métodos foram executados de maneira robusta o suficiente para produzir resultados confiáveis.

Sim.

4. Os métodos estatísticos foram apropriados para analisar os dados, as análises são completas e os dados numéricos são consistentes e plausíveis.

Sim.

5. As tabelas e figuras são bem elaboradas, de fácil compreensão e complementam o texto de forma eficaz, sem redundâncias e com informações precisas.

As ilustrações contendo os mapas coroplético com os resultados do estudo estão de fácil entendimento.

6. As conclusões do estudo são sustentadas pelos resultados e respondem à questão de pesquisa.

Sim.

7. O resumo apresenta com clareza os principais resultados da pesquisa, com números que apoiam as afirmações, e seus métodos esclarecem como tais resultados foram obtidos. A conclusão responde ao objetivo, que é claro e conciso.

O Resumo apresenta clareza nos resultados, nos números. Os métodos foram robustos. A conclusão foi descrita no final da discussão de forma clara.

8. As palavras-chaves são pertinentes ao manuscrito e quando não estão no DeCS são necessárias ao tema.

Todas as palavras chaves estão no DeCs e são pertinentes ao manuscrito.

9. O trabalho apresenta novas perspectivas, conhecimentos ou descobertas relevantes para a área de estudo.

Em um estudo recente é descrito que mais de 60% das hospitalizações por transtornos gastrointestinais da doença de Chagas são de emergência e em 50% delas resultam em procedimento cirúrgico (4). Ocasionando custos ao Estado que impacta na previdência social e a assistência social. Neste estudo pode-se identificar as regiões que são mais acometidas pela doença relacionando com variáveis, como idade, gênero, resultando em parâmetros para intervenções.

Referência retirada do manuscrito:

4. Bierrenbach AL, Quintino ND, Moreira CHV, Damasceno RF, Nunes MDCP, Baldoni NR et al. Hospitalizations due to gastrointestinal Chagas disease: National registry. PLoS Negl Trop Dis. 2022;16(9):e0010796.

10. Os resultados do estudo indicam potencial para aplicação em diferentes contextos e populações, indo além de um interesse puramente local.

Sim, os resultados apresentados são a nível de Brasil.

11. O estudo apresenta resultados completos e conclusivos, indo além de dados preliminares ou descritivos.

Sim, o estudo usou técnicas exploratórias de dados espaciais, nomeadamente a autocorrelação espacial.

12. Os métodos estão bem descritos, permitindo a replicação do estudo por outros pesquisadores e com adesão adequada ao guia de redação do delineamento.

Sim, foram descritas quais técnicas utilizaram e os programas estatísticos.

13. Os procedimentos empregados no estudo indicam cuidado na minimização de vieses, garantindo a validade e confiabilidade dos resultados.

Sim, foram associados os índices I de Moran Global e Local e foram acompanhadas do teste de pseudosignificância.

14. O estudo utiliza instrumentos de coleta de dados previamente validados e com boa confiabilidade.

Sim, a fonte de dados foi o Sistema Único de Informações de Benefícios do Ministério da Previdência Social, privativo, desenvolvido pela Empresa de Tecnologia e Informações da Previdência Social

15. A amostra é representativa da população de interesse e tem tamanho suficiente para gerar resultados estatisticamente robustos.

Foram concedidos 4.661 benefícios no período estudado, porém o uso do banco de dados do Regime Geral da Previdência Social estabeleceu limitação ao estudo, devido incluir apenas indivíduos com doença de Chagas inscritos no sistema previdenciário desse regime ou aqueles que possuíam os requisitos básicos para terem direito aos benefícios assistenciais. Segundo o autor o estudo apresenta baixa taxa de perdas de participantes que não compromete a confiabilidade dos achados.

16. Os autores discutem as implicações práticas ou a importância de suas descobertas e/ou apresentam uma conclusão clara sobre a validade de sua hipótese, com base nos resultados do estudo.

Sim, através dos resultados sinalizam as regiões com maior prevalência da doença, fazem considerações sobre as variáveis estudadas e buscam identificar as causas destas condições apresentadas, sinalizando a importância do estudo para intervenções e estudos longitudinais de pacientes.

17. A terminologia adotada e denominações de variáveis seguem padrão científico e comunicam claramente o sentido pretendido.

Sim.

18. A escrita é de fácil compreensão, com linguagem precisa e objetiva e com boa organização das ideias.

Acredito que a descrição da faixa etária está com o símbolo matemático errado, se a população estudada possui idade de maior e igual a 29 anos e menor e igual a 60 anos, pelo que foi observado nos números apresentados no resultado, então a forma correta de escrever é (29≤ Idade ≤60).

19. O texto é livre de jargões em excesso, construções desnecessárias e vícios de linguagem (excesso de conjunções [ex.: portanto, contudo], hipérboles/exageros, excesso de adjetivos e advérbios, frases longas, clichês, redundâncias, linguagem coloquial ou inacessível).

Sim.

20. Siglas são restritas às consagradas e ao mínimo essencial para fluidez do texto.

Sim.

21. O manuscrito segue as normas gramaticais e ortográficas, incluindo uso de maiúsculas.

Sim.

22. O manuscrito observa as diretrizes do periódico, indicando cuidado dos autores na preparação dos arquivos.

Sim.

23. As referências citadas são adequadas (compatíveis com o trecho a que são atribuídas).

Sim, são adequadas, porém não consegui acessar a referência 27.

24. A lista de referências bibliográficas inclui os trabalhos mais recentes e relevantes e aparenta estar completa.

Sim, apresenta referências atuais relacionadas ao estudo, sendo que das 27 referências bibliográficas do manuscrito, 02 de são do ano de 2024; 01 de 2023; 4 de 2022; 03 de 2021 e 01 de 2020, totalizando 11 referências.

25. As imagens, gráficos e figuras são de alta qualidade, adequadas para publicação e contribuem para a clareza e o impacto visual do trabalho.

Sim.

